# Unravelling the Genetic Landscape of Hemiplegic Migraine: Exploring Innovative Strategies and Emerging Approaches

**DOI:** 10.3390/genes15040443

**Published:** 2024-03-31

**Authors:** Mohammed M. Alfayyadh, Neven Maksemous, Heidi G. Sutherland, Rod A. Lea, Lyn R. Griffiths

**Affiliations:** Centre for Genomics and Personalised Health, Genomics Research Centre, School of Biomedical Sciences, Queensland University of Technology (QUT), Brisbane, QLD 4059, Australia; mohammed.alfayyadh@hdr.qut.edu.au (M.M.A.); n.maksemous@qut.edu.au (N.M.); heidi.sutherland@qut.edu.au (H.G.S.); rodney.lea@qut.edu.au (R.A.L.)

**Keywords:** hemiplegic migraine, sequence variants, bioinformatics, whole exome sequencing

## Abstract

Migraine is a severe, debilitating neurovascular disorder. Hemiplegic migraine (HM) is a rare and debilitating neurological condition with a strong genetic basis. Sequencing technologies have improved the diagnosis and our understanding of the molecular pathophysiology of HM. Linkage analysis and sequencing studies in HM families have identified pathogenic variants in ion channels and related genes, including *CACNA1A*, *ATP1A2*, and *SCN1A*, that cause HM. However, approximately 75% of HM patients are negative for these mutations, indicating there are other genes involved in disease causation. In this review, we explored our current understanding of the genetics of HM. The evidence presented herein summarises the current knowledge of the genetics of HM, which can be expanded further to explain the remaining heritability of this debilitating condition. Innovative bioinformatics and computational strategies to cover the entire genetic spectrum of HM are also discussed in this review.

## 1. Introduction

Migraine is a severe, debilitating neurovascular disorder that is significantly influenced by genetic factors. One prominent clinical feature of migraine is the manifestation of episodes of reversible focal neurological symptoms, including visual, sensory, or speech disturbances called aura [1]. The International Headache Society (ICHD-III, 2018) has classified migraine into two major types based on the existence of aura: migraine with aura (MA), which is less common type, and migraine without aura (MO) [1].

Hemiplegic migraine (HM) is a very rare and severe subtype of MA that is characterised by the presence of motor weakness in one side of the body, called hemiplegia [2]. HM is an early-onset disorder that affects people during the first or second decade of their lives [3]. HM often resembles other complex disorders, such as stroke, which may present a challenge for clinical diagnosis [3,4]. Current research shows that HM is a far less prevalent subtype of MA that affects ~0.01% of European populations [3,5]. HM is split into two classes according to whether family history is involved. The first class is familial hemiplegic migraine (FHM), which can be clinically recognised in patients for whom medical history consists of the presence of at least one first- or second-degree family member with HM. FHM tends to follow an autosomal mode of inheritance, and missense mutations have been robustly implicated in the three known genes, which are *CACNA1A*, *ATP1A2*, and *SCN1A* [6]. The second class is called sporadic hemiplegic migraine (SHM), which can be diagnosed in patients for whom family history lacks the presence of HM [7]. Moreover, SHM can be recognised when de novo mutations exist in the known FHM genes [3,4,8,9].

Genome-wide association studies (GWAS), linkage analysis, positional cloning, and candidate gene studies have been the central focus of migraine research. However, GWAS have mainly investigated common variants. Moreover, research in HM has been on single nucleotide variants without considering the role of larger structural variations. Innovative strategies, such as using gene-based association testing rather than single-variant association testing, employing machine learning to reveal genomic patterns that might be implicated in HM, and investigating the impact of large variations in HM, hold the promise of revealing the actual genetic makeup of this condition.

In this review, we aim to summarise the current knowledge surrounding the genetics of HM. Furthermore, we will propose strategies currently absent in the literature on the genetic architecture of HM, address existing gaps in the literature, and outline future directions.

### 1.1. Epidemiology

#### 1.1.1. Prevalence of Migraine, Including HM

There is a substantial economic and health burden associated with migraines. The prevalence of migraine ranges between 15% and 20% in the general population [10], impacting one billion people globally [11]. According to the World Health Organization, migraine is considered the sixth most disability-causing disorder worldwide [12] and the third most debilitating and disability-causing condition among individuals under 50 years of age [13]. HM, a rare form of migraine, has a prevalence of 0.01% [7]. Adolescents between 12 and 17 experience HM most often [10], with females being affected more frequently than males [7,14]. The severity and frequency of the HM attacks decrease progressively with age [3]. FHM is a rare disorder with an estimated low prevalence rate of 0.003% [5,7,15,16,17]; it follows an autosomal mode of inheritance and is a very rare subtype of MA [3,18]. SHM is not different regarding rarity as it has a 0.002% prevalence rate [10]. In addition to the reduced quality of life among members of societies impacted most by migraine, there is a significant economic burden. In the United States and Europe, the economic burden of managing migraine cases is more than 19 billion dollars, which is expressed as lost productivity and indirect medical costs in the United States [11].

#### 1.1.2. Clinical Features

What distinguishes HM from other forms of migraine is the presence of hemiplegia, which is expressed as weakness or paralysis in one side of the body, involving the loss of voluntary movement and muscle control [1,2]. However, the underlying mechanism that initiates the formation of hemiplegia and aura is not fully understood. Common factors, such as viral infection, physical and emotional stress, and head trauma, have been found to trigger HM [19,20]. The headache symptom, which often manifests as a result of HM, occupies different positions, including unilateral, bilateral, ipsilateral, or contralateral to the side of the weakness [3]. The unilateral weakness is the most common symptom that accompanies HM attacks and is a fundamental sign in the diagnosis of HM. The unilateral weakness can switch sides but rarely occurs bilaterally.

Clinicians diagnose MA by confirming the presence of sensory and visual defects during migraine attacks. The sensory features include numbness, tingling and paraesthesia, while the visual symptoms involve scintillating scotoma and hemianopia. Other accompanying features during the attacks may include seizure, fever, bilateral visual defects, brainstem aura with vertigo, ataxia, hyperacusia, dysarthria, disturbed consciousness, tinnitus, and, in severe cases, coma [3,5,14,21]. In most cases, HM lasts between 20 and 60 min, but sometimes the aura and weakness occur in an abrupt state resembling an ischaemic-like event [22]. However, in severe cases, the consciousness defect and hemiplegia may last for weeks before full recovery occurs [23,24,25,26].

The presence of aura and hemiparesis distinguishes FHM, a heritable form of migraine [6]. However, excluding hemiparesis, the clinical features that accompany the aura in FHM are similar to those of common types of migraine [6]. Most FHM patients experience the clinical features of both MA and MO [27]. Chronic features, such as progressive ataxia and gaze-evoked nystagmus, have been reported to be dependent on the gene involved as they occur in only 60% of FHM type 1 caused by pathogenic variants in *CACNA1A* (FHM1) but rarely in FHM type 2 caused by *ATP1A2* variants (FHM2) [3,22,28,29]. Some patients with HM because of *CACNA1A* and *ATP1A2* mutations have reported developing mental retardation and cognitive disorder following migraine attacks [30,31]. Moreover, specific *CACNA1A* mutations in some children with FHM1 have been linked to cognitive dysfunction associated with vermian cerebellar atrophy [32].

## 2. The Genetic Basis of HM

### 2.1. FHM and the Three Known Genes

The traditional approach to deciphering the complexity of migraine causation involves studying the genetic makeup of families with heritable migraine phenotypes. The underlying assumption in following this approach is that both common and rare monogenic forms of migraine share the same fundamental genetic mechanism that eventually triggers migraine attacks in pedigree members. Therefore, studying genetic mutations in heritable migraine could lead to the discovery of a unifying genetic theory that explains all forms of migraine. 

Linkage analysis identified a higher load of mutations linked to genes involved in synaptic signalling in the central nervous system (CNS) that differentiate patients with FHM from those with common forms of migraine [15]. To date, three genes, *CACNA1A*, *ATP1A2*, and *SCN1A*, have been established to have a causal relationship with FHM. These three genes are responsible for encoding ion transporters. FHM is, therefore, classified into FHM1 if mutations occur in the *CACNA1A* gene, FHM2 if mutations occur in the *ATP1A2* gene, and FHM3 if mutations occur in the *SCN1A* gene [3,33,34]. Although mutations in the *CACNA1A*, *ATP1A2*, and *SCN1A* genes cause FHM, their occurrence is rare [15,35,36,37].

Adding to the complexity of understanding the interplay between the human genome and migraine development is the fact that the involvement of mutations in the three known FHM genes is not always established in patients with FHM; this supports the speculation that there might be unknown genes involved in FHM [38]. For example, a recent study found that genetic variants in the *CACNA1I* gene might contribute to the aetiology of HM [39]. A Danish study on FHM revealed that only 14% of the affected individuals had mutations in the three known genes. In comparison, a large study conducted in Finland reported mutations in only 9% of 45 families with FHM [5,40]. Studies concluded that the three known FHM genes account for only 7–14% of FHM cases, supporting the notion that other loci might be responsible for developing this debilitating condition [5,39,41,42].

From a clinical perspective, distinguishing FHM caused by each of the three implicated genes is challenging due to overlapping symptoms. Patients with mutations in the three different FHM genes also differ in presenting phenotypes, even when they have mutations in the same gene or from one family unit with the same mutations [34,43,44,45]. These findings further support the possibility that other genes might be involved in FHM or other environmental or genetic factors that confound the relationship between the three known genes and FHM [42]. To understand this complex disorder, mouse models have been used as an experimental system as they provide a unique gateway into the mechanisms that underly brain dysfunctions that lead to migraine development.

#### 2.1.1. FHM Due to Mutations in the *CACNA1A* Gene

The first gene that underlies FHM development is *CACNA1A*. This gene is located on chromosome 19p13 and regulates the function of the α subunit of Cav2.1 voltage-gated calcium channels in the central nervous system [6,33,34,35]. Cav1.2 channels are primarily distributed in the nervous system and abundant in most of the brain’s active area [46,47]. These channels serve mainly as triggers for neurotransmitter release in the central synapses and neuromuscular junction [48,49].

More than 30 mutations in the *CACNA1A* gene have been identified in patients with FHM and SHM [3]. Commonly reported mutations in this gene are in the Clinvar database Table 1. Most of these mutations are missense variants and deletions [50]. Mutations in the *CACNA1A* gene account for 50–75% of FHM cases and are suggested to initiate a mechanism disrupting the function of Cav2.1 channels [35,51,52]. Extensive evidence indicates that FHM1 mutations result in a gain of function of Cav2.1 channels, leading to abnormal glutamate neurotransmission and enhanced neuronal hyperexcitability [34,35,53,54]. To investigate this finding further, researchers suggest that the disruption impacts these channels’ opening and activation processes, leading the gates of these channels to open easily under low voltages [46,54]. As a result, the unbalanced excitatory–inhibitory mechanism at the synaptic level increases susceptibility to cortical spreading depression (CSD) [55,56]. In addition to the theory of CSD, the complexity of the effects of FHM1 mutations on susceptibility to HM is shaped by other factors. Studies investigating whether sex modifies the relationship between mutations in the *CACNA1A* gene and migraine have shown that female sex hormones enhance susceptibility to CSD [53]. These studies have found that female hormones contribute to the list of factors that modify the effect of mutations on different forms of migraine disorders, including HM [34,57].

Mouse models of FHM1 with Ki knock-in (KI) mutations, specifically R192Q and S218L, revealed increased susceptibility to CSD and change in the balance of excitation/inhibition [56,59,60,61,62], alteration in the plasticity of synapsis [63], and change in pain signalling [64,65]. These two *CACNA1A* mutations, R192Q and S218L, have been generated in mouse models [55,66]. The R192Q mutation is often associated with a less severe form of the disease [67]. In contrast, animals with the S218L mutation often exhibit combined phenotypes, including seizures and cerebellar ataxia [68], similar to those developed by patients with FHM1 mutations. These mouse models of FHM1 mutations R192Q and S218L have shown increased calcium influx in the neuro system, hyperexcitatory neurotransmitter release in the cortex [18,59,68,69], and increased susceptibility to CSD [55,66,70,71,72]. Although sex and stress hormones modify the increased susceptibility to CSD in KI mice with R192Q and S218L mutations [73,74], concerning CSD frequency and severity [68], mice with R192Q mutations have, additionally, displayed metabolic alterations as a consequence of CSD initiation [75]. The change in metabolites such as lysine and pipecolic acid signifies an increase in the GABAergic neurotransmission following excitation as a compensation procedure [61,75]. Moreover, the consequences of CSD in mice with R192Q mutation extended to changes in the repertoire of peptides and metabolites in the brain [76]. Studies have concluded that the R192Q and S218L mutations are associated with gain of function [55,66]. Furthermore, the development of change in the excitation/inhibition balance [56,59,60], modification to the signalling of pain in the trigeminal nuclei, and alteration in the plasticity of the synapsis are mainly the consequences of these mutations [63].

#### 2.1.2. FHM Due to Mutations in the *ATP1A2* Gene

Another important gene implicated in FHM is *ATP1A2*. Common mutations associated with this gene are in Table 2. The *ATP1A2* gene is located on chromosome 1q23.2. In contrast to *CACNA1A*, which regulates ion channels, *ATP1A2* encodes the α2 subunits of the sodium–potassium ATPases pump [6,36]. The genetic influence of the *ATP1A2* gene involves regulating the work of the α2 subunits of the Na+/K+ ATPase ion transport pump that underlies the process of electrochemical activity in the central nervous system, heart and skeletal cell membranes [34,77]. In early ages, the work of the *ATP1A2* gene is mainly related to neurons, but its expression involves glial cells in adulthood. 

Mutations in the *ATP1A2* gene are associated with 20% of FHM cases [36,78,79,80]. More than 80 genetic mutations exist in the *ATP1A2* gene, the most mutated gene causing FHM [81]. Most of these mutations are missense [34], while the others, classified as deletions, are found in patients with SHM [82]. Mutations in the *ATP1A2* gene lead to alteration in the pump’s sensitivity to potassium intake [83,84], disruption of the potassium/sodium replacement rate [85], and the production of dysfunctional proteins [77,86,87]. Alteration in the sensitivity of the pump plays a crucial role in the development of HM. Disruptions in the pump function lead to clearing the cell membrane of K+ ions by admitting them into the cell and releasing the Na+ ions outside the cell, creating a Na+ gradient across the cell membrane necessary to reuptake glutamate [88]. Moreover, in addition to the impact on the pump’s sensitivity, mutations in this gene disrupt the clearance of glutamate and potassium and, consequently, increase susceptibility to CSD [89,90].

Mouse models have been generated to study the effects of FHM2 mutations, although few *ATP1A2* mutations have been studied for their functional effects [6,91]. Further supporting the overarching understanding that mutations in FHM genes influence CSD, mutations in the FHM2 gene are likewise associated with increased susceptibility to CSD in KI mice [89]. Various mutations in *ATP1A2* affect CSD via different mechanisms. The T345M mutation in *ATP1A2* reduced potassium intake, while R689Q and M731T mutations were associated with increased potassium intake and reduced exchange rate [83,92]. At the cellular level, the L764P and W887R mutations in *ATP1A2* led to the generation of nonfunctional proteins [18,93]. In addition to generating nonfunctional proteins, the W887R and G301R mutations disrupted the level of glutamate taken by glial cells [69,89,94] and induced CSD [55,66,89,95]. W887R mutant mice have shown a decreased K+ and glutamate clearance level in synapses, leading to an increased chance of CSD [89,90]. 

Moreover, because of the W887R mutation, behavioural changes concerning pain responses have also been observed in mice [89]. In heterozygous KI mice with the FHM *ATP1A2* G301R mutation, changes in behaviour involved an increased level of anxiety, fear, and depression, combined with a decreased level of mobility [94]. Similar to FHM1, sex plays a crucial role in KI mouse models of FHM2. Abnormal behaviours, which include features of obsessive-compulsive disorder, have been noticed primarily among female KI mice with FHM2 mutations [94]. Interestingly, progestin treatment reversed these abnormal behaviours to the normal state [94]. Heterozygous female mice with FHM2 mutations showed increased susceptibility to CSD [61,89] due to cortical astrocytes’ low clearance of K+ and glutamate [90]. It has been suggested that the level of glutamate released in females is also influenced by the female cycle hormone, possibly explaining the abnormal behavioural and emotional state in FHM2. It seems that the functional consequences of FHM2 mutations are numerous. Still, the overarching effect is the loss of normal function of the potassium–sodium turnover, possibly predicting glial cells’ decreased glutamate and potassium intake.

#### 2.1.3. FHM Due to Mutations in the *SCN1A* Gene

The *SCN1A* gene is located on chromosome 2q24.3. This gene encodes the α1 subunit of the neuronal voltage-gated sodium channels. These channels control the production and propagation of excitation actions in the neuronal cells [49,96]. These channels also control the permeability of sodium ions of the GABA interneurons of the central nervous system [97]. Mutations in this gene are mainly missense and account for approximately less than 5% of FHM families [37,52]. Common mutations in this gene are in Table 3. An autosomal dominant mode of inheritance is how mutations of this gene are expressed [62,98,99]. The complexity of *SCN1A* mutations is manifested in the mechanism by which they lead to both gain and loss of function of Nav1.1 channels [100]. Studies have shown that the gain of function of Nav1.1 channels can lead to elevated excitability of cortical interneurons [47,101], while the loss of function can lead to the initiation of epilepsy syndrome [102,103]. The consequence of the gain of function in the neuronal channels is the elevation in the release of glutamate and susceptibility to CSD [104,105]. *SCN1A* mutations have been discovered in both pure FHM families and FHM patients with other disorders, such as epilepsy and intermittent daily blindness [106,107]. One *SCN1A* mutation is Q1489K. This mutation was found in three German families with FHM [37]. Another *SCN1A* mutation, L1649Q, was identified in a North American family with FHM [107].

A recent KI mouse model using the FHM3 human mutation L1649Q has been generated [108]. This model showed an increased action of neuronal firing in the interneurons because of the gain of function caused by the L1649Q mutation [61,108]. The Q1489K mutation in this gene has also been suggested to cause a gain of function, leading to increased neuronal excitability and neurotransmitter release [95]. Additionally, the FHM3 mutation L263V is associated with spontaneous episodes of CSD [109]. These CSD events involved the motor and visual systems resembling clinical features reported in humans.

Regarding the responses of mice with mutations in this gene, which are not significantly different from other forms of FHM, FHM3 mutant mice displayed increased susceptibility to more frequent CSD events [108]. In general, heterozygous mice with FHM3 mutations experienced seizures and deaths, with a higher likelihood of epilepsy due to the low level of sodium intake in the GABAergic inhibitory interneurons. Further data on FHM3 concerning behavioural changes is of great importance and may be different due to the effect of FHM3 mutations on the interneurons as opposed to the consequences of FHM1 and FHM2 mutations [108].

Overall, *CACNA1A* and *ATP1A2* mutations have functional consequences, including impairments in the cognitive ability of patients and, in some cases, intellectual disability following multiple attacks [30,31,77,110]. Cognitive dysfunction has been reported in 50% of children aged 3–18 years [32]. FHM1 gene mutations in Cav2.1 disrupt and consequently increase glutamate release from the cortical neurons, resulting in CSD propagation. Mutations in the FHM2 gene disrupt the potassium–sodium pump function, reducing potassium and glutamate uptake. FHM mutations associated with the Nav1.1 sodium channel can predict in vivo hyperexcitability and neurotransmitter release. It appears that mutations in the three known FHM genes increase the release of potassium and glutamate in the synaptic cleft, enhancing the probability of CDS [111].

#### 2.1.4. Other Potential Genes Associated with FHM

Other genes, *PRRT2*, *SLC1A3*, and *SLC4A4*, have been previously implicated in some HM cases, although their involvement in HM remains controversial. The *PRRT2* gene, discovered in 2012, was proposed as a possible fourth gene for FHM [112,113]. It has been suggested that the *PRRT2* gene could be a potential fourth gene that might be linked to the development of HM [62,114,115,116]. Despite frequent findings of mutations in *PRRT2* among HM patients [115,117], accounting for less than 5% [118,119], its role is considered complex. *PRRT2* regulates the neurons’ voltage-gated calcium channels and extracellular glutamate release [34,120]. Mutations in this gene might lead to an elevated presynaptic vesicle release and, consequently, hyperexcitability [117]. Another suggestion was made that this gene has a role in sodium channels, as patients with *PRRT2* mutations responded effectively to carbamazepine, the antiepileptic drug that blocks sodium channels [112,121]. Although the consideration of *PRRT2* as a fourth gene for HM is common knowledge among researchers, many suggested that the complexity of the mechanism via which *PRRT2* influences HM and the phenotypic heterogeneity seen with *PRRT2* mutations forces the hypothesis that it might be working as a modifying factor [118]. These observations suggest that the *PRRT2* gene does not specifically cause HM, and the most likely conclusion is that there might be other genetic variants involved in the few cases of HM that carry *PRRT2* mutations [119]. Therefore, mutations in the *PRRT2* gene are unlike mutations in the main three FHM genes as they may not be enough to cause HM in a Mendelian fashion.

*SLC1A3* is another gene implicated in HM, even though the evidence for its causal relationship is not definitive. This gene encodes the amino acid transporter EAAT1, which transmits glutamatergic release in the neurons. Mutations in *SLC1A3* were found in HM patients and can cause episodic ataxia type 6 [122,123]. The missense mutation P290R in *SLC1A3* was discovered in one patient who suffered from episodic ataxia, seizures, and hemiplegia [122]. Another missense *SLC1A3* mutation T387P that disrupted the potassium binding to EAAT1 has been reported in a patient with HM, leading to the notion that EAAT1 might be implicated in HM. However, this patient’s father had the same mutation without showing HM symptoms [123]. Currently, evidence for causality predicated on isolated case reports in which clinical evaluation is not without suspicion appears insignificant [122,123]. Therefore, these findings are insufficient for this gene to be considered an HM gene [122].

*SLC4A4* is another gene that may contribute to HM. The *SLC4A4* gene is crucial in encoding the sodium bicarbonate cotransporter NBCe1. Two HM patients were found to have a *SLC4A4* mutation (S982NfsX4), suggesting a potential causal relationship [124]. Common mutations associated with *SLC1A3* and *SLC4A4* are presented in Table 3.

Furthermore, *CACNA1I* and *CACNA1H* may be implicated in HM [39,125]. Patients with HM have been found to have an increased burden of missense variants in these two genes, further supporting the hypothesis that the genetic architecture of HM extends beyond the currently known genetic area for HM.

### 2.2. SHM

The sporadic form of HM shares the same clinical features as the FHM form, except for the absence of a family history of HM [2,33]. Approximately 35% of FHM cases are recognised clinically as manifesting SHM mainly because of the lack of a family history of HM [7,62]. In addition to the lack of family history with HM, SHM can be recognised when de novo mutations are present in the known FHM genes [3,4,8,9]. For example, de novo mutations of the FHM2 gene *ATP1A2* are commonly reported in SHM cases [34,77]. A complex inheritance mechanism shaped by a combination of genetic and environmental factors might explain the initiation of SHM [3,62,126]. Many findings support the understanding that other unknown genes might form the genetic foundation of both FHM and SHM, given that both disorders share similar clinical features [3]. Such a suggestion is not without a strong basis. A Danish study on SHM found that 92 out of 100 patients had no mutations in the known FHM genes [127]. A Finnish study did not find FHM genetic mutations in 201 patients diagnosed with SHM [41]. It appears that a sophisticated polygenic mechanism involving many genetic variants might be implicated in SHM [4], and a similar mechanism might likewise explain the development of FHM cases, especially when there are no mutations in the three FHM known genes [4].

## 3. Innovative Approaches That Offer the Potential to Explain the Remaining Heritability of HM

### 3.1. Learning from the Legacy of GWAS

Fundamentally, two scientific perspectives on the mechanism underlie the relationship between the frequency of variants and complex diseases. The first perspective suggests that many variants are rare, with significant effects on the general population, collectively leading to common diseases [128]. On the contrary, the other view asserts that a small number of high-frequency variants with small individual effects act together to cause common diseases [129]. Both are likely relevant to many disorders, including HM.

Genome-wide association studies (GWAS) have significantly contributed to understanding the genetic architecture of many traits by revealing many novel and vital associations [130]. Most associations discovered by GWAS are between common variants and diseases [131]. These studies have mainly investigated the relationship between disease and common variants with a minor allele frequency (MAF) greater than 5%, discovering more than 2000 variants [132]. These variants explained various diseases, including the role of autophagy in Crohn’s disease [133], predisposition to obesity and the role of the CNS, and macular degeneration and ageing [134]. The original hope from GWAS has been that as the number of common variants significantly associated with complex traits increases, common variants will cluster, and ultimately, such variants will implicate biological pathways. 

However, GWAS, with their focus on variants with frequencies of <1–5%, have only been able to explain 5–10% of the heritability of disease [135]. GWAS associations did not explain most of the genetic variance that underlies many complex traits [136,137]. This suggests that GWAS have failed to cover the genetic spectrum of common variants causing complex diseases. A counterargument suggests that common variants might tend to spread broadly if a sufficient number of individuals are genotyped [138]. Therefore, the hope of GWAS to explain the genetic foundation of complex traits is becoming less realisable. 

The remaining heritability may be partly explained by rare, highly penetrant variants [139]. Variants with MAF of less than 1% are considered rare variants. Suppose we examine the basis of the evolutionary theory, then deleterious variants, although rare, can seriously alter protein generation [140,141]. Strong evidence indicates that variants less frequent in the general population contribute to complex diseases [142,143]. For example, recent GWAS revealed that rare variants contributed to common migraine [144].

### 3.2. Exome Studies

Many genome sequencing designs have their strengths and weaknesses. This section focuses on the importance of whole exome design and discusses the most common rare variant association tests. 

As the cost of whole exome sequencing (WES) technologies becomes more affordable, the number of whole exome studies is growing. Knowing which variant is relevant to disease causation in common variant studies has been particularly challenging. However, identifying true associations could be simplified by focusing on rare variants in functional genomic regions. The recent development in sequencing techniques combined with the decrease in sequencing cost has enabled exome studies to focus on rare variants in exonic regions. Studies including the National Heart, Lung, and Blood Institute (NHLBI) exome project, the T2D-GENES project, and the UK10K project are all exome studies focused on rare variants and were successful in enriching the dbSNP database with millions of rare variants [141,145]. 

Exome sequencing is a very effective sequencing design that aims to investigate the 1–2% of the human genome that controls protein production [146]. Many disorders with a Mendelian inheritance pattern have been linked to causal variants discovered by exome studies. For example, causal variants for Miller syndrome [147], Kabuki syndrome [148], late-onset Alzheimer’s [149], and low-density lipoprotein cholesterol [150], have been identified through exome studies. Despite the limitation of missing the noncoding regions of the human genome, exome studies remain an essential approach for exploring the rare variant makeup of complex disorders.

### 3.3. Investigating Large Structural Variations

Although structural variations (SVs) and copy number variations (CNVs) account for the most variance in the human genome, they remain under-investigated. Replication studies in the human genome have shown that most genetic variants considered CNVs reside in genomic regions of at least 1 kb in size [151,152]. Population studies have identified thousands of SVs in the human genome that are >5 kb [153,154]. Rare gains or losses in CNVs are responsible for approximately 15% of human neurodevelopmental diseases due to disruption in the dosage of many genes [155]. Conditions like kidney dysfunction, autism and congenital heart disease are linked to large CNVs and SVs, either de novo or inherited. The functional impact of CNVs and SVs ranged from disruption of gene expression, as is the case of the *CHRNA7* gene and migraine, to complex disease development [156]. Unfortunately, these classes of sequence variation have been underrepresented in human genetic studies at all levels. The potential of expanding our understanding of the human genome relies significantly on studying CNVs and SVs, as they play a crucial role in disease initiation. 

#### 3.3.1. CNVs

Individuals typically inherit two copies of the DNA sequence from their parents, but through poorly understood mechanisms, specific DNA sequences may exhibit CNVs, becoming one, three, or even more copies. CNVs are an important type of sequence variation that may or may not involve a gene. Generally, CNVs are defined as a 1 kb genetic sequence that displays a change in copy number relative to the reference genome. CNVs are sequence variations, including splicing, deletions, and duplications of segments of the DNA [156]. The human genome project led to the understanding that genetic materials have been lost and gained in the human genome. Technological advances, including the design of sophisticated bioinformatic tools, have made detecting CNVs possible. Common tools used to detect CNVs are in Table 4.

It has been suggested that many mechanisms underlie the development of CNVs. The repairment processes include the homology-directed and nonhomologous DNA strands undergoing meiotic recombination and erroneous replications [176]. However, the relative contribution of each of these mechanisms to the generation of CNVs is not known. 

When the frequency of CNVs is below 1%, they are considered rare; otherwise, they are common. The relationship between CNVs and disease is not fully understood. However, many cancers have been linked to either an increase or decrease in the number of genetic regions involving genes [156]. Large CNVs have been found to cause generalised epilepsy [177], children’s developmental delays [178], and cardiac defects [179]. Other disorders, such as brain malformations and different forms of seizures, were also associated with CNVs [180]. Complex conditions, such as intellectual disability, schizophrenia, and autism, have been directly linked to CNVs. Changes in the copy number of particular genes might cause disease if these genes are involved in dose-sensitive functions [181]. For example, a change in the copy number of the genetic region that involves the *CHRNA7* gene is highly effective in neuropsychiatric diseases with severe consequences [182]. Gains in copy number have been associated with autism, anxiety and other mental conditions [183].

Interestingly, CNVs have been found to modify the effect of rare mutations on complex trait development [184]. Moreover, gains and losses in copy numbers have been found to cause migraines [156]. However, the mechanism was unclear as either the gain or loss changed the gene dosage and, consequently, its function. The relationship between intronic and exonic regions of the genome is still complex. CNVs may start outside the exonic regions but can extend to the gene regions. When copy number gains involve a segment of a gene sequence, the remaining coding sequence of the gene becomes disrupted. Variants outside the exonic regions may still impact the process by which genes encode protein. 

#### 3.3.2. SVs

SVs involve many sequence alterations, including inversions, translocations, deletions, insertions, and other rearrangements. Research in SVs and disease has identified many inverted genomic sequences. The sequence inversion that involved the factor VIII gene has been found in 40% of individuals with haemophilia [185]. Other inversions that impacted the emerin gene have been linked to Emery-Dreifuss muscular dystrophy [186]. However, the overall contribution of inversions, as a type of SVs, to disease in the general population remains unknown. Another type of SVs is translocations or cryptic rearrangements. 

Translocations play a role in developing different phenotypes that range from causing the Wolf-Hirchhorn syndrome to dysmorphic features [187]. Four SVs are associated with complex traits: a deletion of 20 kb in size upstream of the Immunity-Related GTPase Family M (IRGM) gene explained Crohn’s disease [188], another deletion of 45 kb upstream of the NEGRI gene with body mass index [189]; a 32 kb deletion with psoriasis [190]; and a 117 kb deletion to with osteoporosis [191]. Although the evidence is not strong that SVs correlate directly to phenotypic consequences, their influence on gene dosage and expression and other environmental factors can cause or predispose individuals to genetic diseases [192,193].

The fundamental approach for detecting SVs and CNVs involves leveraging signatures that are the outcome of mapping discordance between a sample and the reference genome. Four main approaches have been the focus of research to detect SVs and CNVs; the read-pair method investigates the distance and orientation of paired ends, the depth method for detecting gains and losses in copy number, the split-read method considers whether alignments cover SV breakpoints, and de novo reassembly of contigs before comparison with the reference genome [194,195,196].

Various SV and CNV callers have been developed, including PEMer [197], BreakDancer [198], and CNVnator [170], which all depend on at least one of these four approaches. This, however, limits the detection of SVs and CNVs. Other detection tools, such as Manta, LUMPY, DELLY, and GenomeSTRiP [199], combine different signatures and approaches, thereby improving sensitivity and mitigating issues single-approach algorithms face. 

Another reason not to focus solely on single-variant association tests is that CNVs have been found to cover large regions of the human genome, including coding sequences. Single variants will never reveal the extent to which a genomic region is associated with complex traits. CNVs have been found to alter 12.5% of gene transcripts and disrupt the coding sequence of 5.5% of mRNAs [176]. For example, one study showed that 32% of all trait-associated variants fell within regions that contained CNVs [176]. Those variants were significantly correlated with both CNVs and 22 traits. It has been suggested that common CNVs (MAF > 5%) could explain some of the remaining heritability that GWAS failed to decipher in complex traits. 

### 3.4. Rare Variant Association Testing

Association tests performed by GWAS have decreased utility in rare variant investigations since such tests are primarily designed for common variants with a higher frequency in the general population. Moreover, single rare-variant association tests are inherently underpowered due to the challenges of sample size in complex traits. Investigating the role of rare variants requires a large sample size since such variants have a very low frequency in the general population. To overcome the limitations of tests used by GWAS, many attempts have been made to design alternative methods for testing rare variants over the past few years [200,201,202,203]. 

These alternative methods include collapsing and/or burden tests and distribution-based analyses. They are also gene- or region-based tests, considering that multiple variants can be grouped. The premise of collapsing methods is hinged on the idea that a group of rare variants from different genomic regions can act together to cause a common trait. The higher the number of variants with criteria such as minor allele frequency below 1% and a higher impact on protein function in a genomic region, the greater the likelihood that this region is conducive to disease development. 

Collapsing tests typically combine information about genetic variants from various sites within a predefined genetic region or gene into a single variable. One association test is the gene-based collapsing test. The idea is that cases and controls are compared for the relative distribution of a qualifying variant, which is a variant that satisfies the selection criteria in a certain gene. Each gene is investigated for the number of cases and controls that carry at least one qualifying variant [204]. Due to the increasing technical ability to deal with variants that are rare in the population, collapsing methods are gaining importance in explaining complex traits. Such tests have gained power over the past few decades because they also increase statistical power [202,205,206]. 

#### 3.4.1. Single-Variant Association-Based Test

This analysis typically employs a linear regression model to investigate a potential relationship between a single variant and a trait within a case-control design [207]. Thousands of causative variants have been identified using this approach. Single variant tests have the potential to uncover rare variants if sample or variant effect sizes are sufficiently large. One large GWAS study with approximately 8000 individuals identified variants impacting insulin processing through this testing approach [208]. However, this type of test remains ineffective in exploring associations between rare variants and traits, as very large sample sizes or many variants with large effects are required to overcome the lack of power. 

#### 3.4.2. Region- or Gene-Based Tests

In this type of test, aggregated information from multiple variants at different sites is given one score or weight. The combined information is then tested for association with the disease of interest. Here, we will focus on the regression-based methods that allow users to adjust for covariates. Common association tests are presented in Table 5.

#### 3.4.3. Burden Tests

Many statistical methods that deal with associations between rare variants and traits have been developed, including the kernel-based approach (kernel-based adaptive cluster (KBAC)) and the sequence kernel association test (SKAT). SKAT and burden tests are the most used for their flexibility and ability to maintain high testing power. SKAT, a flexible and computationally efficient statistical regression model, allows for the adjustment of covariates. Within a regression model, burden tests assign a single score to combined information from various variants at various genomic sites, considering factors like MAF, with variants having a frequency threshold. In this type of analysis, every additional rare variant increases disease risk [209]. Other tests assume the presence of a common genetic structure that underlies both common and rare variants in disease causation. One such method is the combined multivariate and collapsing method (CMC), which is an approach designed specifically for case-control data. The CMC combines information about common and rare variants in a single gene or genomic region. This approach, then, assigns a single score to the aggregated information and tests for association with the trait of interest using Hoteling’s *t*-test [205]. The fundamental assumption in burden tests is that all variants have one direction of effect with the trait [210,211]. These methods are less powerful when variants have a bidirectional relationship and when there are many non-causal variants. These methods have been used extensively, proving the importance of considering each rare variant association testing unique and that more emphasis should be placed on tailoring study designs to these methods [212].

#### 3.4.4. Variance-Component Tests

Analysing the distribution of the effects of multiple variants within a certain genomic region is another rare variant association approach. This approach is used by SKAT [213], the C-alpha test [210], and the sum of the squared score (SSU) test [214]. These methods have been designed specifically to address the issue of directionality and the unstable number of causal variants as they use mixed models. However, these tests are less powerful when genomic regions have more causal variants and/or variants with no bidirectional effects. 

To address potential issues arising from using either the variance-component test or burden tests, the p values from both approaches can be combined. Derkach et al. [215], Sun et al. [216], and Lee et al. [211] developed the omnibus test SKAT-O. They combined both tests using a Fisher statistic model. SKAT-O takes advantage of SKAT and burden tests, but such tests lose power when the number of variants associated with the trait of interest is small [217,218]. As an alternative, the aggregated Cauchy association test (ACAT) takes into account the small number of causal variants and increases the testing power by combining and transforming variant *p*-values to be Cauchy variables [219]. Although SKAT-O is more powerful than either test, it can be less powerful if the underlying assumption of either is true.

Overall, the single-variant association methods are less powerful than gene- or region-based association tests. However, gene- or region-based tests can be weakened when only a few variants within a genomic region are associated with the trait, when the number of variants that have no effect is large and when causal variants are infrequent. Another limitation of the gene-based collapsing approach is the differences in genetic sub-region tolerance for the presence of missense variants. Studies show that disease-causing variants often reside within intolerant sub-regions. However, there are two approaches to deal with this issue. Either collapsing directly on the sub-regions of the genes or including the missense intolerance as another filter when selecting qualifying variants [220,221].

### 3.5. Annotation of Sequence Variants

Due to the significant volume of sequencing data generated by high-throughput platforms, the need to reduce the total number of variants to a functionally relevant subset is growing. Annotating sequence variants is a crucial process that influences the association of genetic variants with phenotypic changes. The annotation aims to predict the impact of sequence variants on gene products and protein function [222,223,224]. Annotation of sequence variants depends on the set of transcripts and software used. Three primary sources of transcripts can be the basis of annotation, including Ensembl [195], RefSeq [196], and UCSC [225]. The RefSeq dataset is regularly updated to encompass all possible and observed transcripts and gene models. The Ensembl dataset provides transcript information, including the CCDs [226,227], Havana [228], Vega [229], Gencode [230], and the Gencode [231]. Software designed for this purpose is required to annotate variants.

### 3.6. Annotation Tools

The Annotate Variation Tool (ANNOVAR) and Variant Effect Predictor (VEP) are among the most widely used annotation tools. Both annotate sequence variants for further variant prioritisation and filtration. ANNOVAR is a newly developed tool for annotating single nucleotide variants (SNVs) and short INDELs [232]. This software has been designed to annotate SNVs and short INDELs so that identifying a subset of variants that impact gene function the most is easily achievable. ANNOVAR offers gene-, region-, and filter-based annotations. For gene-based annotations, ANNOVAR can identify the impact of SNVs and CNVs on protein function. Users can use ANNOVAR to annotate sequence variants using a gene list from RefSeq, UCSC, ENSEMBL, GENCODE, or AceView genes. For region-based annotations, ANNOVAR can recognise genomic regions from which sequence variants have emerged. For example, ANNOVAR can identify conserved regions, predicted transcription factor binding regions, GWAS hits, DNAse hypersensitivity sites and segmental duplication regions. For filter-based annotation, ANNOVAR can identify sequence variants reported previously. For example, if sequence variants have been reported in the dbSNP database, ANNOVAR can recognise such variants. It can also annotate sequence variants with information from different datasets, including the 1000 Genome Project, Exome Aggregation Consortium (ExAC), and gnomAD. Moreover, ANNOVAR can predict functional consequences of sequence variants using SIFT, PolyPhen, LRT, MutationTaster, MutationAssessor, Fathmm, MetaSVM, MetaLR, GERP++ < 2, or CADD > 10.

VEP is another annotation tool designed and developed by ENSEMBL [233]. It annotates sequence variants in coding and non-coding genomic regions. It has been used by GWAS [234] and other projects such as the 1000 genomes [141], and ExAC [235]. VEP annotates input variants with information about their impact on protein, transcription, and regulatory regions. VEP adds annotations regarding allele frequencies and associated phenotypes for known sequence variants previously reported.

### 3.7. Machine Learning

As illustrated above, recent research has focused on rare variants. However, due to the nature of such investigation, the issue of obtaining large sample sizes is one of the challenges faced by this type of research. A large sample size is required to maximise the impact of rare variants on disease or to ascertain whether there are associations between rare variants and complex traits [236]. As this is often not possible, studies have adopted modern innovative computational strategies, such as machine learning (ML), to predict associations and the impact of rare variants on disease [237,238]. It has been suggested that using machine learning models to explore genetic risk patterns in genomic data derived from WES while focusing on rare variants would explain more missing heritability [239]. 

What makes machine learning models more suitable in genotype–phenotype association is their ability to handle small datasets where the number of genetic risk factors exceeds the number of samples being tested. Traditional statistical testing imposes stringent calculations, allowing only a few variants to pass. However, the nature of genetic variation research requires more efficient computation to allow for more complex interactions, as unpassed variants might confer disease risk [240]. 

Machine learning can be divided into two main classes, including supervised and unsupervised models [241]. The supervised approach can be either a regression-based or tree-based model. Their names drive the design of both classes. Supervised machine learning models are appropriate when the outcome of interest is known and is the goal of prediction. In contrast, the unsupervised approach does not require a predetermined investigative association. 

Supervised models are increasingly utilised in the field of genetics, where research aims to explore genetic patterns that might infer a complex trait. Supervised models can be used for classification purposes in a case-control setting when the outcome is a binary variable or for regression formulation when the outcome is a continuous variable. These models learn from a source of truth or training data where the outcome is known, and associated predictors are predetermined [242,243]. In contrast to the goal of classical statistical methods that search for potential associations to explain variation at the population level, supervised models prioritise explaining variation at the individual level [244,245,246]. These models have the power to deal with complex interactions among genetic variants [247,248] that are often missed by single locus testing [249]. Classical models assume the existence of independent variants, which often leads to poor prediction power where epistasis effects, gene–gene interactions, linkage disequilibrium, and nonrandom relationships among variants at different loci exist; these models lack such assumptions [250,251].

Furthermore, GWAS has employed the polygenic risk scoring (PRS) system to test for association between a group of potentially causal variants and a trait. This system does not assume interactions among variants. Unlike the classical approach of exploring associations between individual variants and complex traits, machine learning considers the possibility of existing interactions among variants of small effects that usually escape the statistical significance of conventional statistical methods [252].

Machine learning models, such as random forest (RF) and gradient boosting, allow for complex interactions [253]. RF has been applied in many studies. Recently, a study identified interactions among environmental risk predictors for myocardial infarction as a binary outcome and coronary artery calcification as a quantitative variable [253,254]. In addition, several SNVs that have a causal relationship with rheumatoid arthritis have been identified using random forest (RF), Bayesian network analysis (BNA), and artificial neural networks (ANN) machine learning models. Many SNVs have been found to contribute to the development of rheumatoid arthritis using these models [255]. 

Classical tools designed to predict pathogenicity include sorting intolerant from tolerant (SIFT) [256], polymorphism phenotyping v2 (PolyPhen2) ref. [223], combined annotation dependent depletion (CADD) [257], and consensus deleteriousness score (CONDEL) [258]. Although these tools have advanced the field of differentiating between disease-causing and benign variants, they are still not powerful enough in their predictions [259]. 

A fundamental stage that is considered the foundation of most models that achieve high predictive power in genetic variant association is feature selection and regularisation. In this stage, the subset of variants that are most predictive of the trait of interest are identified. Feature selection aims to reduce data dimensionality and exclude variants without an independent role in prediction [239]. Research has used three feature selection methods in genetic predictors, including the embedded, wrappers, and filters [243,253,260,261]. Regularisation restricts the number of predictors the predictive model uses by excluding irrelevant patterns in the training data to avoid overfitting the model.

Studies have adopted penalised regression approaches such as the least absolute shrinkage and selection operator (lasso) [262] and ridge regression [263] for feature selection. Feature selection is integral to building an effective prediction machine learning model. Although these approaches control the selection of genetic features that might be efficiently used in predictive modelling, their usage has been limited. Traditionally, variants correlated with the disease of interest are selected based on prior knowledge about their impact [242,243,260,264]. However, recent research emphasises the importance of using machine learning models to generate a subset of data on features or genetic variants that might drive disease risk. These methods model the relationship between a group of variants with different effects sized against the outcome while accounting for interactions. These methods, however, have not been designed to capture nonlinear interactions between SNVs and between SNVs and diseases [265]. A study used support vector machines (SVM) and ridge logistic regression to ascertain variants that might implicate type 1 diabetes. They used a fivefold cross-validation (CV) process as simple cross-validation may not eliminate the overfitting and highly optimistic modelling issue and achieved high predictive power with an area under the curve (AUC = 0.9) [252]. 

However, machine learning models are not immune to limitations. It is often the case that these models are prone to overfitting and intensive computation. Predictive models, by design, rely heavily on the size of the training dataset, the genetic basis of the trait of interest, and the presence of additional information about individuals, such as family history [264,266,267,268,269]. One challenge in constructing machine learning models is generating a practical evaluating framework to determine their predictive ability [270]. It must be noted that considering the generalisability of the prediction model on new datasets and successful passing through the cross-validation process is crucial. Despite the advances achieved through machine learning in genome-wide data, this practice remains underrepresented [261,264]. On the contrary, the utilisation of machine learning in other types of genetic studies, such as genome-wide gene expression profiles, has been extensive. 

### 3.8. Criteria to Be Considered When Selecting Variants within a Case-Control Design

The number of qualifying variants tends to be inflated in older people, necessitating adjustment for age at sample collection. Moreover, the number of qualifying variants tends to be inflated in underrepresented populations. Therefore, extra care should be given to samples derived from under-represented populations for the lack of accurate frequencies of variants from such samples within their populations. Another important criterion that should be considered is the impact of variants on protein change. Such variants include in-frame deletion or insertion (INDELs), missense variants, canonical splice sites and protein-truncating variants. Many tools have been designed to capture variants, including CADD, PolyPhen-2, SIFT, REVEL, and PrimateAI.

Matching cases with controls should be addressed. Coverage of sequence should be harmonised between cases and controls. Moreover, ideally, controls should lack the disease of interest. The possibility of a high contamination rate should be considered, and regions with low capture rates or samples with low coverage should be removed. Qualifying variants cancel each other when performing ultra-rare association testing, so one individual from each pair of relatives should be excluded. Ultra-rare variants are very unique to the sample being tested [271]. MAF should be calculated as an internal measure of frequency for all cohorts, including cases and controls, to avoid bias. The same filter should be used to calculate internal MAF and external MAF (gnomAD). The ultimate goal is to provide evidence of homogeneity between cases and controls [271]. Additionally, to reduce background variation due to sequencing quality variance, contamination, confounding factors between cases and controls, and unknown factors, background variation behind every gene should be minimised to allow true risk variants that are clinically relevant to manifest in the test.

## 4. Conclusions

To date, research in the genetics of HM has implicated three main genes in HM, including *CACNA1A*, *ATP1A2*, and *SCN1A*. The *CACNA1A* gene encodes the subunit α1 of the neuronal voltage-gated Cav2.1 Ca2+ channel, while the *SCN1A* and *ATP1A2* genes encode the subunits of Nav1.1 Na+ and glial Na+K+ ATPases channels, respectively. Mutations in the three genes disrupt ion channels and transporters’ functions. The *CACNA1A* mutations increase glutamate release due to enhancing calcium influx at the presynaptic level. *ATP1A2* mutations disrupt Na+ transport and consequently change the level of synaptic glutamate *SCN1A* mutations increase discharge frequency, which in turn elevates the synaptic glutamate level. The mutual consequence of sequence variation in these three genes seems to enhance the glutamate level at the synaptic cleft of cells. As a result, abnormal intense and frequent firing by the neurons associated with glutamatergic synapses initiates the increased susceptibility to CSD, which underlies the formation of migraines with aura. Of note, the continuing discovery of new causative mutations in and outside the three known genes could increase our understanding of what distinguishes HM from common forms of MA.

While GWAS have been the conventional method for exploring the genetic basis of migraine, there is a growing interest in studying CNVs and SVs due to recent discoveries. However, few studies have attempted understanding CNVs and SVs [193,272,273]. As far as we are aware, no studies have extensively examined the role of CNVs and SVs in migraine forms, including HM and gene-based burden/collapsing in HM research. In exploring the genetic variation underlying migraine, research has primarily focused on the effects of SNVs [274]. Consequently, such research must employ a detailed filtration process to eliminate all possible false-positive variants. This filtration process depends on datasets containing repositories of SNVs, CNVs, and SVs. Research indicates that, on average, each individual has over 1000 sequence variations, including CNVs [275]. 

HM is a debilitating and uncommon disease that CNVs and SVs may cause. Numerous studies suggest that these sequence variations are rare, but their combined impact is quite significant. Therefore, failing to consider the role of CNVs and SVs when studying the genetic basis of a disease like HM will not wholly explain its heritability [136]. Although next-generation sequencing (NGS) technology can identify these types of sequence variations at the sequence level, utilising innovative computational methods, such as machine learning and gene- or genomic region-based association testing within a whole exome design to investigate their association with the disease, is critical for understanding their diversity and function in complex traits, including HM.

## Figures and Tables

**Table 1:** The ClinVar [58] most reported mutations in the *CACNA1A* gene associated with HM.

Gene	Mutation	ID	Functional Consequences	Caused Disorders
*CACNA1A*	R192Q	rs121908211	Gain of function, increased calcium influx and susceptibility to CSD and metabolic alterations.	Migraine, FHM
	S218L	rs121908225	Gain of function, increased calcium influx in the neuro system and led to hyper excitatory neurotransmitter release in the cortex, and subsequently elevated susceptibility to CSD	FHM is implicated in epilepsy. HM, SHM, different types of seizures and cerebellar ataxia
	E1015K	rs16024	Altered Cav2.1 channels function and have inactivation properties, leading to a gain of function	HM, MA
	I1512T		n/a	FHM
	R2157G	rs554393704	n/a	FHM
	T501M	rs121908240	Gain of function, altered channel activation/inactivation process and promoted CSD.	FHM, progressive ataxia and EA2
	R583Q	rs121908217	Disrupted channel activation/inactivation process.	FHM, SHM, progressive ataxia and EA2
	T666M, T665M	rs121908212	Gain of function via leading to a reduction in recovery after inactivation and increased calcium influx	FHM, SHM, Progressive Cerebellar Ataxia and cerebellar dysfunction
	I1811L	n/a	Altered P/Q-type calcium currents and accelerated recovery from inactivation	FHM, cerebellar ataxia
	V714A	rs121908213	Accelerated channel recovery from inactivation	FHM and cerebellar symptoms, including ataxia
	D715E	rs121908218	Disrupted channel inactivation process and glutametric release	FHM
	E2080K	rs752513542	n/a	SHM
	P2479L	rs764648125	n/a	SHM
	H2481Q	rs539546830	n/a	SHM
	Y1384C	rs121908219	Loss of function in Cav2.1	FHM, SHM, coma, cerebellar ataxia, and cerebellar atrophy
	I710T	n/a	n/a	FHM has been found to cause epilepsy, cerebral oedema, fatal coma, and seizure
	V713M, V714M	n/a	n/a	FHM, Developmental and epileptic encephalopathy, EA2
	Q1673fs, Q1674fs, Q1676fs, Q1679fs	n/a	n/a	FHM, Developmental and epileptic encephalopathy, EA2, Spinocerebellar ataxia type 6
	A1507T, A1508T, A1511T	n/a	n/a	FHM, Developmental and epileptic encephalopathy, EA2, Migraine, familial hemiplegic, Spinocerebellar ataxia type 6
	K771, K772, K775	n/a	n/a	Developmental and epileptic encephalopathy, 52, EA2, Migraine, familial hemiplegic, Spinocerebellar ataxia type 6
	V1811I, V1808I, V1809I, V1814I	n/a	n/a	Developmental and epileptic encephalopathy, 52, EA2, Migraine, familial hemiplegic, Spinocerebellar ataxia type 6
	C281fs	n/a	n/a	Developmental and epileptic encephalopathy, 52, EA2, Migraine, familial hemiplegic, Spinocerebellar ataxia type 6
	R1779, R1780, R1782, R1785	n/a	n/a	Developmental and epileptic encephalopathy, 52, EA2, Migraine, familial hemiplegic, Spinocerebellar ataxia type 6
	T1355N, T1356N, T1359N	n/a	n/a	Developmental and epileptic encephalopathy, 52, EA2, Migraine, familial hemiplegic, Spinocerebellar ataxia type 6
	c.3990-2A>C	n/a	n/a	Migraine, familial hemiplegic
	R1667P, R1672P, R1666P, R1669P	n/a	n/a	Developmental and epileptic encephalopathy, 52, EA2, Migraine, familial hemiplegic, Spinocerebellar ataxia type 6
	F550fs, F551fs	n/a	n/a	Developmental and epileptic encephalopathy, 52, EA2, Migraine, familial hemiplegic, Spinocerebellar ataxia type 6
	E1212, E1211, E1215	n/a	n/a	Migraine, familial hemiplegic
	T1355N, T1356N, T1359N	n/a	n/a	Developmental and epileptic encephalopathy, 52, EA2, Migraine, familial hemiplegic, Spinocerebellar ataxia type 6
	V1811I, V1808I, V1809I, V1814I	n/a	n/a	Developmental and epileptic encephalopathy, 52, EA2, Migraine, familial hemiplegic, Spinocerebellar ataxia type 6
	c.978+1G>C	n/a	n/a	Migraine, familial hemiplegic
	L1344P, L1345P, L1348P	n/a	n/a	Developmental and epileptic encephalopathy, 52, EA2, Migraine, familial hemiplegic, Spinocerebellar ataxia type 6
	V1806A, V1807A, V1809A, V1812A	n/a	n/a	Developmental and epileptic encephalopathy, 52, EA2, Migraine, familial hemiplegic, Spinocerebellar ataxia type 6
	D1316E, D1317E, D1320E	n/a	n/a	Developmental and epileptic encephalopathy, 52, EA2, Migraine, familial hemiplegic, Spinocerebellar ataxia type 6
	G700E, G701E	n/a	n/a	Developmental and epileptic encephalopathy, 52, EA2, Migraine, familial hemiplegic, Spinocerebellar ataxia type 6
	c.2017-2034del	n/a	n/a	Developmental and epileptic encephalopathy, 52, EA2, Migraine, familial hemiplegic, Spinocerebellar ataxia type 6
	L617S, L618S	n/a	n/a	Developmental and epileptic encephalopathy, 52, EA2, Migraine, familial hemiplegic, Spinocerebellar ataxia type 6
	S218P	n/a	n/a	Developmental and epileptic encephalopathy, 52, EA2, Migraine, familial hemiplegic, Spinocerebellar ataxia type 6
	I1707T, I1708T, I1710T, I1713T	n/a	n/a	Developmental and epileptic encephalopathy, 52, EA2, Migraine, familial hemiplegic, Spinocerebellar ataxia type 6

n/a—not available.

**Table 2:** The ClinVar [58] most reported mutations in the *ATP1A2* gene associated with HM.

Gene	Mutation	ID	Functional Consequences	Caused Disorders
*ATP1A2*	D178N	n/a	Loss of function	FHM, increased risk of epilepsy and mental retardation
	P979L	rs121918615	Loss of function	FHM, increased risk of epilepsy and mental retardation
	R1007W	rs746795369	Disruption of K+ levels in the CNS	FHM and increased susceptibility to epilepsy
	R593W	rs886039530	Reduced rate of the pump activity	FHM
	V628M	rs1553245659	Reduced rate of the pump activity	FHM
	W887R	rs28933399	Generation of non-functional proteins, disruption of the level of glutamate taken by glial cells, decreased clearance of K+ in the synapses and initiation of CSD	FHM and altered pain responses
	T345M	n/a	Reduced potassium intake	FHM
	T345A	rs121918613	lower affinity for potassium	FHM
	R689Q	rs28933401	Increased potassium intake led to a reduction in the exchange rate at the cellular level.	FHM, benign familial infantile convulsion
	M731T	rs28933400	Increased potassium intake led to a reduction in the exchange rate at the cellular level.	FHM
	G301R	rs121918612	Disrupted the level of glutamate taken by glial cells	FHM
	E700K	n/a	n/a	FHM and coma
	L764P	rs28933398	Generation of non-functional proteins	FHM
	R383H	rs765909830	n/a	FHM
	E825K	n/a	Loss of function of the sodium-potassium pump	FHM and types of seizures
	A606T	rs1414742926	Loss of function of the sodium-potassium pump	FHM and epileptic seizures
	M745I	n/a	n/a	SHM
	R879Q	rs761597771	n/a	SHM
	R879W	n/a	n/a	SHM
	Y9N	rs55858252	n/a	SHM
	R383H	rs765909830	n/a	SHM
	G615R	rs770053423	Complete loss of function of the pump	FHM and neurological features
	I240fs	n/a	n/a	FHM
	P786L	n/a	n/a	FHM
	I286fs	n/a	n/a	FHM
	S779N	n/a	n/a	Alternating hemiplegia of childhood 1
	Y1009	n/a	n/a	Epilepsy, Familial hemiplegic migraine
	R834Q	n/a	n/a	FHM
	G366V	n/a	n/a	FHM
	C581	n/a	n/a	FHM
	T263M	n/a	n/a	FHM
	R1002Q	n/a	n/a	FHM, Migraine, familial hemiplegic
	V628M	n/a	n/a	FHM
	A606T	n/a	n/a	FHM
	G855R	n/a	n/a	FHM
	G715R	n/a	n/a	FHM
	R421	n/a	n/a	FHM
	R834	n/a	n/a	FHM
	V191M	n/a	n/a	FHM
	T376R	n/a	n/a	FHM
	T376M	n/a	n/a	FHM
	I286T	n/a	n/a	FHM
	R548H	n/a	n/a	FHM
	P979L	n/a	n/a	FHM
	T345A	n/a	n/a	Migraine, familial hemiplegic
	G301R	n/a	n/a	Familial hemiplegic migraine
	T378N	n/a	n/a	FHM, Alternating hemiplegia of childhood 1
	W887R	n/a	n/a	Migraine, familial hemiplegic
	L764P	n/a	n/a	Migraine, familial hemiplegic
	M731T	n/a	n/a	Migraine, familial hemiplegic
	T378N	n/a	n/a	FHM, Alternating hemiplegia of childhood 1
	D718N	n/a	n/a	FHM
	G855E	n/a	n/a	FHM
	R421	n/a	n/a	FHM
	R937H	n/a	n/a	Inborn genetic diseases, FHM
	D812H	n/a	n/a	FHM
	R1002Q	n/a	n/a	FHM, Migraine, familial hemiplegic
	T263M	n/a	n/a	FHM
	Y1009	n/a	n/a	Epilepsy, FHM
	R937fs	n/a	n/a	Alternating hemiplegia of childhood, Migraine, familial hemiplegic
	I630L	n/a	n/a	Migraine, familial hemiplegic
	M829V	n/a	n/a	FHM
	T368M	n/a	n/a	FHM
	R202W	n/a	n/a	FHM

n/a—not available.

**Table 3:** The ClinVar [58] most reported mutations in the *SCNIA*, *SLC1A3*, and *SLC4A4* genes are associated with HM.

Gene	Mutation	ID	Functional Consequences	Caused Disorders
*SCN1A*	L1649Q	n/a	Associated with a gain of function and increased action firing in the interneurons	FHM
	L263V	n/a	Increased susceptibility to CSD	FHM, epilepsy and other seizures
	Q1489K	n/a	Overall, it causes a gain of function, leading to increased neuronal excitability and release of neurotransmitters. Some results showed a loss of function.	FHM
	Q1489H	n/a	n/a	FHM and elicited repetitive daily blindness
	F1499L	n/a	n/a	FHM and elicited repetitive daily blindness
	F1774S	n/a	The overall gain of function	SHM
	T1174S	n/a	Switch between loss and gain of function, but there is an overall loss of function impact, deceleration of recovery from fast inactivation.	FHM and epileptic seizures
	L263Q	n/a	n/a	FHM and epileptic seizures
	L1624P	n/a	Gain of function via decreasing fast inactivation predicts hyperexcitability.	FHM
	I1498M	n/a	n/a	FHM
	F1661L	n/a	n/a	FHM
	L1670W	n/a	Gain of function via modifying the channel gate properties	FHM
	R1613G, R1614G, R1630G, R1631G, R1642G, R828G	n/a	n/a	Migraine, familial hemiplegic
	L1328P, L1312P, L1329P, L1340P, L1311P, L526P	n/a	n/a	Migraine, familial hemiplegic
	L1634S, L1635S, L1652S, L1663S, L1651S, L849S	n/a	n/a	Migraine, familial hemiplegic
	N1350I, N564I, N1349I, N1378I, N1366I, N1367I	n/a	n/a	Migraine, familial hemiplegic 3, Generalized epilepsy with febrile seizures plus, type 2, Severe myoclonic epilepsy in infancy
*SLC1A3*	P290R	n/a	n/a	Episodic ataxia, seizures, and hemiplegia
	T387P	n/a	Disruption of the binding of potassium to EAAT1	HM
*SLC4A4*	S982NfsX4	n/a	Loss of function	HM
	L522P	n/a	Loss of function due to disruption of synaptic pH	SHM and episodic ataxia

n/a—not available.

**Table 4:** The most used tools for CNV discovery.

Tool	Platform	Description	License	Credit Publication
CNVkit	Python	Detection and visualisation of CNVs. Mainly for WES data	Free	The National Institutes of Health [157].
AbsCN-seq	R	Detection of CNVs in NGS data	Free	The National Institutes of Health [158].
ABSOLUTE	R	Detection of CNVs and multiplicity of mutations in NGS data	Free for non-commercial use	The National Cancer Institute, the National Human Genome Research Institute, the National Institute of Health, National Research Service Award [159].
aCNViewer	Docker, R, Python	Visualisation of CNVs and loss of heterozygosity in tumour samples	Free	Community of developers [160].
Affy6CNV	R, Perl, C++	Pipeline for calling CNVs from Affymetrix genotyping data.	Free	Community of developers [161].
AluScanCNV2	R	Calling CNVs from NGS data	Free	Private developers, University grants Hong Kong SAR [162].
CNVcaller	Python, Perl	Detection of CNVs in large populations.	Free	The National Natural Science Foundation of China, the National Thousand Youth Talents Plan [163].
CNVannotator	Web-based	Annotation of CNVs	Free	The National Institutes of Health, the Robert J. Kleberg, Jr. and Helen C. Kleberg Foundation, Ingram Professorship Funds [164].
CNValidator	Python	Evaluation of the correctness of copy number calls.	Free	The National Institutes of Health (NIH) [165].
CNV-seq	R, Perl	Estimation of CNVs, a statistical approach for CNVs assessment	Free	National University of Singapore [166].
CNV-RF	Perl, R	Detection of CNVs in NGS data	Free	thya0003_at_umn.edu [167].
CNVeM	C	Detection of CNVs	Free	The National Toxicology Program and National Institute of Environmental Health Sciences [168].
CNVer	C, C++	Detection of CNVs	Free	cnver_at_cs.toronto.edu [169].
CNVnator	C++	Discovery and characterisation of CNVs	Free	The US National Institutes of Health [170]
CNVphaser	Perl	Detection of CNVs	Free for non-commercial use	Grants-in-Aid for Scientific Research (jsps.go.jp) [171].
CNVtest	R	Testing CNVs association based on log-ratio data of SNP arrays	Free	The US National Institutes of Health [172].
ExomeCNV	R	Detection of CNVs and loss of heterozygosity	Free	The US National Institutes of Health [173].
exomeCopy	R	Detection of CNVs from exome data	Free	European Union’s Seventh Framework Program [174].
PennCNV2	C++	Detection of CNVs from SNP arrays and NGS data	Free	kai_at_openbioinformatics.org [175].

**Table 5:** Common gene- or genetic region-based association tests.

Gene- or Region-Based Tests				
Test	Design	Strengths	Limitations	Tools
Burden tests: ARIEL, CAST, CMC, MZ, WSS	They all collapse genetic variants into single scores	Powerful and accurate when most variants are causal and have the same direction of effect	Less powerful when the number of causal variants is small and/or some variants increase disease risk and others decrease disease risk	EPACTS, GRANVIL, PLINK/SEQ, RVTESTS, SCORE-SEQ, SKAT, VAT
Adaptive burden tests: aSum, Step-up, EREC test, VT, KBAC, RBT	Use weights from the adaptive burden test.	More robust compared to other tests that aggregate information, use predetermined thresholds	Require intense computational power	EPACTS, KBAC, PLINK/SEQ, RVTESTS, SCORE-SEQ, VAT
SKAT, SSU, C-alpha test	Test variance of genetic effects	Contrary to burden tests, they are powerful when the number of causal variants is small, and most variants do not have the same direction of effect as the trait.	They lose power when most variants are causal and their effects have the same direction.	EPACTS, PLINK/SEQ, SCORE-SEQ, SKAT, VAT
Joint tests: SKAT-O, Fisher method, MiST	Burden tests are combined.	Reserve power regardless of the number of causal variants and the direction of effect	Power decreases when the underlying assumption of either test is true. The Fisher method requires intense computation.	EPACTS, PLINK/SEQ, MiST, SKAT

## Data Availability

No new data were created or analysed in this study. Data sharing is not applicable to this article.
